# Drug repositioning of mevalonate pathway inhibitors as antitumor agents for ovarian cancer

**DOI:** 10.18632/oncotarget.20046

**Published:** 2017-08-07

**Authors:** Yusuke Kobayashi, Hiroyasu Kashima, Yohan Suryo Rahmanto, Kouji Banno, Yu Yu, Yusuke Matoba, Keiko Watanabe, Moito Iijima, Takashi Takeda, Haruko Kunitomi, Miho Iida, Masataka Adachi, Kanako Nakamura, Kosuke Tsuji, Kenta Masuda, Hiroyuki Nomura, Eiichiro Tominaga, Daisuke Aoki

**Affiliations:** ^1^ Department of Obstetrics and Gynecology, Keio University School of Medicine, Tokyo, Japan; ^2^ Department of Pathology, Johns Hopkins University School of Medicine, Baltimore, MD, United States of America; ^3^ Department of Obstetrics and Gynecology, Shinshu University School of Medicine, Nagano, Japan; ^4^ School of Pharmacy, Curtin Health Innovation Research Institute, Curtin University, Perth, Western Australia

**Keywords:** ovarian cancer, drug repositioning, statin, bisphosphonate, mevalonate pathway

## Abstract

Drug repositioning is an alternative strategy redirecting existing drugs for new disease. We have previously reported an antitumor effect of statins, antidyslipidemic drugs, on ovarian cancer *in vitro* and *in vivo*. In this study, we investigated the antitumor effects of other mevalonate pathway inhibitors and the mechanism of the antitumor effect from a metabolic perspective.

The effects of inhibitors of the mevalonate pathway on tumor cell growth were evaluated *in vitro*. Bisphosphonates that inhibit this pathway are commonly used as antiosteoporotic drugs, and antitumor effects of the bisphosphonate were examined *in vitro* and *in vivo*. Metabolites in SKOV3 ovarian cancer cells were analyzed before and after lovastatin treatment, using capillary electrophoresis-mass spectrometry.

All mevalonate pathway inhibitors showed concentration-dependent inhibitory effects on tumor cell growth. Particularly marked effects were obtained with inhibitors of farnesyltransferase and geranylgeranyltransferase. The bisphosphonate was also shown to have an antitumor effect *in vivo*. The expression of autophagy marker LC3A/3B was increased in cells after treatment. In metabolomics analysis, lovastatin treatment increased the metabolites involved in the tricarboxylic acid cycle while reducing the metabolites associated with glycolysis. Also it decreased glutathione and resulted to work with chemotherapeutic agents synergistically.

Inhibition at any point in the mevalonate pathway, and especially of farnesyl pyrophosphate and geranylgeranyl pyrophosphate, suppresses growth of ovarian cancer cells. Inhibition of this pathway may induce autophagy, cause a shift to activation of the tricarboxylic acid cycle and enhance susceptibility to chemotherapy. Drug repositioning targeting mevalonate pathway for ovarian cancer deserves consideration for clinical application.

## INTRODUCTION

The incidence and mortality of epithelial ovarian cancer have remained unchanged for 20 years, and ovarian cancer is the second most common gynecological cancer worldwide [1]. The five-year survival rate of epithelial ovarian cancer is less than 50% due to difficulties in early detection and rapid disease progression [2, 3]. The outcomes of progressive or recurrent epithelial ovarian cancer are particularly poor; therefore, there is a need to develop new therapeutic agents. Clinical trials to find new drugs were expected to progress after completion of the human genome project. However, candidate drugs found after much research effort and cost may still cause unpredictable adverse events in clinical practice due to difficultly in understanding human pharmacokinetics. Consequently, conventional drug development has not led to launch of new drugs, and a new approach is needed. With this background, drug repositioning has emerged as a new concept in which an existing drug is used for a different disease. Such a drug is already known to be safe and to have appropriate pharmacokinetics in humans, and thus can be used for another disease with markedly less research time and cost compared to conventional drug development [4].

Statins are antidyslipidemic drugs that inhibit hydroxymethylglutaryl coenzyme A (HMG-CoA) reductase in the upstream part of the mevalonate pathway and reduce cholesterol levels in blood [5]. These drugs have pleiotropic effects including antiinflammation, vasodilation and inhibitory effects on vascular remodeling such as coagulation and fibrinolysis, leading to prevention of coronary artery disease, heart failure and arrhythmia [6]. Recent epidemiological studies have also suggested antitumor effects for carcinomas [7–10], but there is no evidence of the utility of statins for ovarian cancer. We have shown that lovastatin has an inhibitory effect on growth of ovarian cancer cells *in vitro*, and antitumor effects such as delayed tumorigenesis and suppression of tumor progression *in vivo* [11]. It was also reported that simvastatin inhibited tumor growth in the syngeneic model and reduced cell migration *in vitro,* resulted to exhibit anti-metastatic and anti-tumorigenic effects in ovarian cancer [12, 13]. In this study, we focused on the potentially important role of the mevalonate pathway in progression of ovarian cancer. We examined whether an inhibitor of this pathway might serve as an antitumor drug for ovarian cancer through drug repositioning and we examined the potential mechanism of antitumor effect from a metabolomic perspective.

## RESULTS

### Induction of autophagy and suppressed cell growth by inhibition of the mevalonate pathway

**Figure 1:**
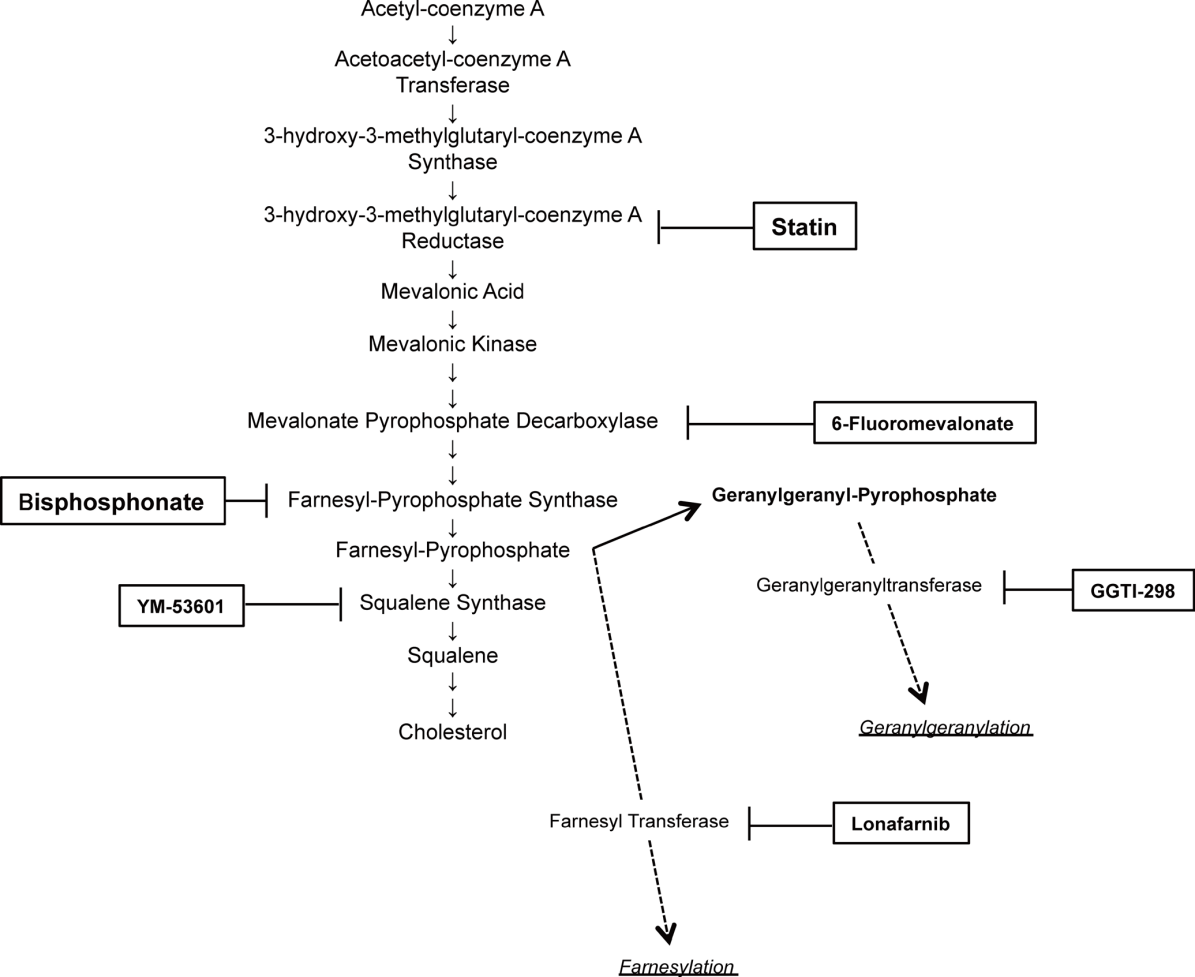
Selected schema of the mevalonate pathway with inhibitors used in this study

**Figure 2:**
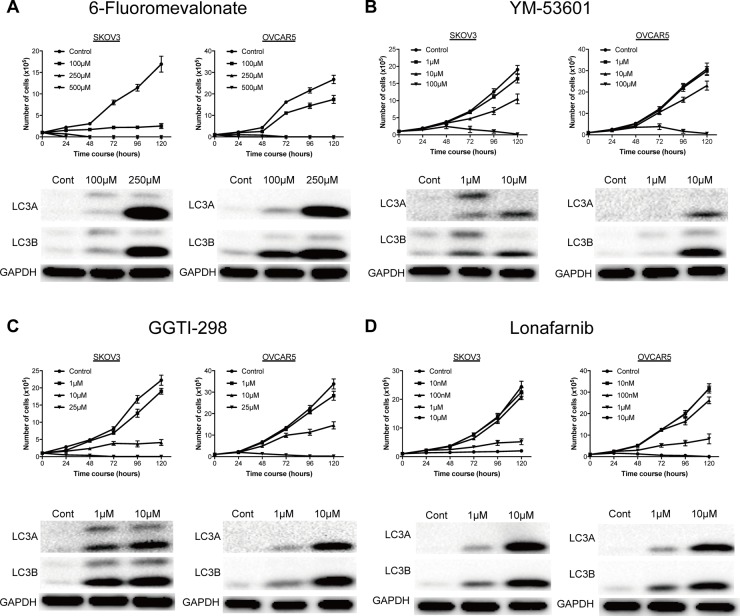
A representative inhibitors on the mevalonate pathway significantly inhibited cell proliferation of ovarian cancer and induced autophagy (**A**) 6-Fluoromevalonate, inhibitor for mevalonate-pyrophosphate decarboxylase, significantly inhibited the cell proliferation and induced the expression of autophagy marker. (**B**) YM-53601, inhibitor for squalene synthase, significantly inhibited the cell proliferation and induced the expression of autophagy marker. (**C**) Lonafarnib, inhibitor for farnesyl transferase, significantly inhibited the cell proliferation and induced the expression of autophagy marker. (**D**) GGTI-298, inhibitor for geranylgeranyl transferase, significantly inhibited the cell proliferation and induced the expression of autophagy marker. Viable cells were measured at 24-hour intervals over a 120-hour period. Data are presented as the mean ±SD (*n* = 3).

We have shown previously that statin inhibition of HMG-CoA reductase in the mevalonate pathway has an antitumor effect on ovarian cancer [11]. In the current study, to determine which step of this pathway is involved in growth of ovarian cancer, we first examined the effects on cell growth of several inhibitors of the pathway: 6-fluoromevalonate, which inhibits mevalonate pyrophosphate decarboxylase; YM-53601, which inhibits squalene synthase; Lonafarnib, a farnesyltransferase inhibitor that blocks farnesylation with farnesyl pyrophosphate; and GGTI-298, a geranylgeranyltransferase inhibitor that blocks geranylgeranylation with geranylgeranyl pyrophosphate (Figure 1). Lonafarnib is being investigated in a human trial as a potential treatment for progeria and chronic hepatitis delta viral (HDV) infection. These agents were added to culture media of SKOV3 and OVCAR5 cells to examine effects on cell growth. All agents inhibited cell growth in a concentration-dependent manner, but Lonafarnib and GGTI-298, which inhibit protein prenylation in a branch of the mevalonate pathway, had inhibitory effects on cell growth at lower doses than 6-fluoromevalonate and YM-53601, which inhibit the main pathway (Figure 2). Vacuolation was found in ovarian cancer cells that were incubated with inhibitors (data not shown), suggesting autophagy. Expression of the autophagy markers LC3A and LC3B was induced by all inhibitors in a concentration-dependent manner, indicating that autophagy was induced by inhibition of all steps in the mevalonate pathway (Figure 2).

### Potential drug repositioning of an antiosteoporotic bisphosphonates

**Figure 3:**
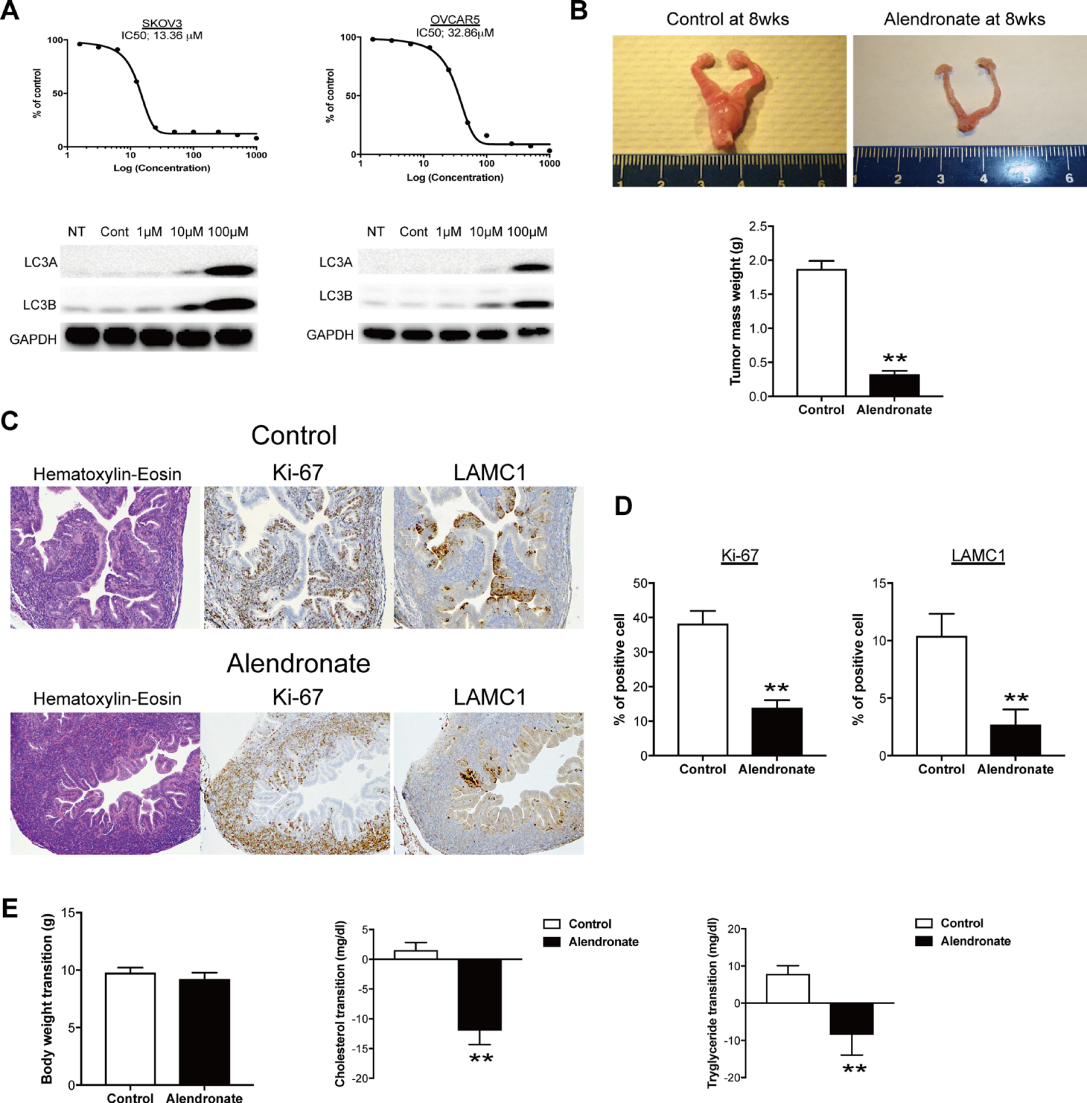
Bisphosphonate-mediated anti-tumor effect *in vitro* and *in vivo* (**A**) IC50 with alendronate significantly inhibited the ovarian cancer cell proliferation and induced the expression of autophagy marker. (**B**) Alendronate administration leads to a significant decrease in ovarian mass in mogp-TAg mice as compared to controls (**p < 0.01). (**C**) Representative images of Hematoxylin-Eosin, Ki-67 and LAMC1 staining on tissue sections from fallopian tubes of mogp-TAg mice. (**D**) Summary of Ki-67 and LAMC1 staining results. Bar graphs depict the percentage of Ki-67–positive or LAMC1–positive epithelial cells among total fallopian tube epithelial cells per section. In each experimental group, data were collected from 10 representative sections from each mouse. **p < 0.01. (**E**) Body weight and serum levels of cholesterol and triglyceride in mogp-TAg mice models. Oral administration of 15 mg/Kg alendronate in the mogp-TAg mice reduced serum levels of cholesterol and triglyceride (**p < 0.01), but did not affect body weight at this dose.

Since inhibition of the mevalonate pathway at many steps had an inhibitory effect on growth of ovarian cancer cells, we examined whether drug repositioning targeting this pathway might be effective for drugs other than statins. Bisphosphonates are commonly used antiosteoporotic drugs worldwide. These drugs inhibit farnesyl pyrophosphate synthase in synthesis of hydrophobic substances such as farnesol and geranylgeraniol that are needed to maintain the function of osteoclasts, which results in reduced osteoclast activity and decreased bone resorption (Figure 1). The effect of alendronate, a second-generation bisphosphonate, on growth of SKOV3 and OVCAR5 cells was examined. Alendronate exhibited concentration-dependent inhibition of growth of these cells (Figure 3A). Similarly to incubation with other inhibitors, vacuolation was found in cells in a time-dependent manner (data not shown) and concentration-dependent expression of LC3A and LC3B was induced by alendronate (Figure 3A).

The potential antitumor effect found *in vitro* was next examined *in vivo* using transgenic ovarian cancer model mice. The mogp-TAg transgenic mouse is a genetically engineered mouse model that expresses the SV40 large T antigen driven by the oviduct glycoprotein 1 promoter [14]. The mogp-TAg mice develop spontaneous serous tubal intraepithelial carcinoma (STIC), the precursor lesion of most ovarian cancers, and uterine stromal sarcoma at 6 to 7 weeks of age [15]. Mice were treated with alendronate (15 mg/kg) or control vehicle beginning at 3 weeks of age, and were euthanized at 8 weeks to evaluate the tumor burden. Treatment with alendronate significantly reduced the total tumor mass in the female reproductive tract, based on the organ weight (Figure 3B). The proliferative activity with Ki-67 and quantification of STICs with STIC-associated marker, laminin C1 [11, 16] were evaluated in tissue section by immunohistochemistry. The proliferative activity with Ki-67 was significantly decreased in the alendronate-treated mice compared with that of the control group (Figure 3C and 3D). The rate of laminin C1-positive tubal epithelial cells in alendronate-treated mice was also significantly reduced compared with that of the control group (Figure 3C and 3D). At the end of the study, plasma levels of cholesterol and triglycerides were significantly reduced in mice treated with alendronate compared with controls (Figure 3E). The alendronate treatment was well tolerated by the mice, and there was no effect on body weight measured at the endpoint (Figure 3E). Collectively, alendronate induced autophagy and had an inhibitory effect on cell growth *in vitro* and an antitumor effect *in vivo*.

### Mevalonate pathway inhibition prevents the Warburg effect and activates the tricarboxylic acid cycle

**Figure 4:**
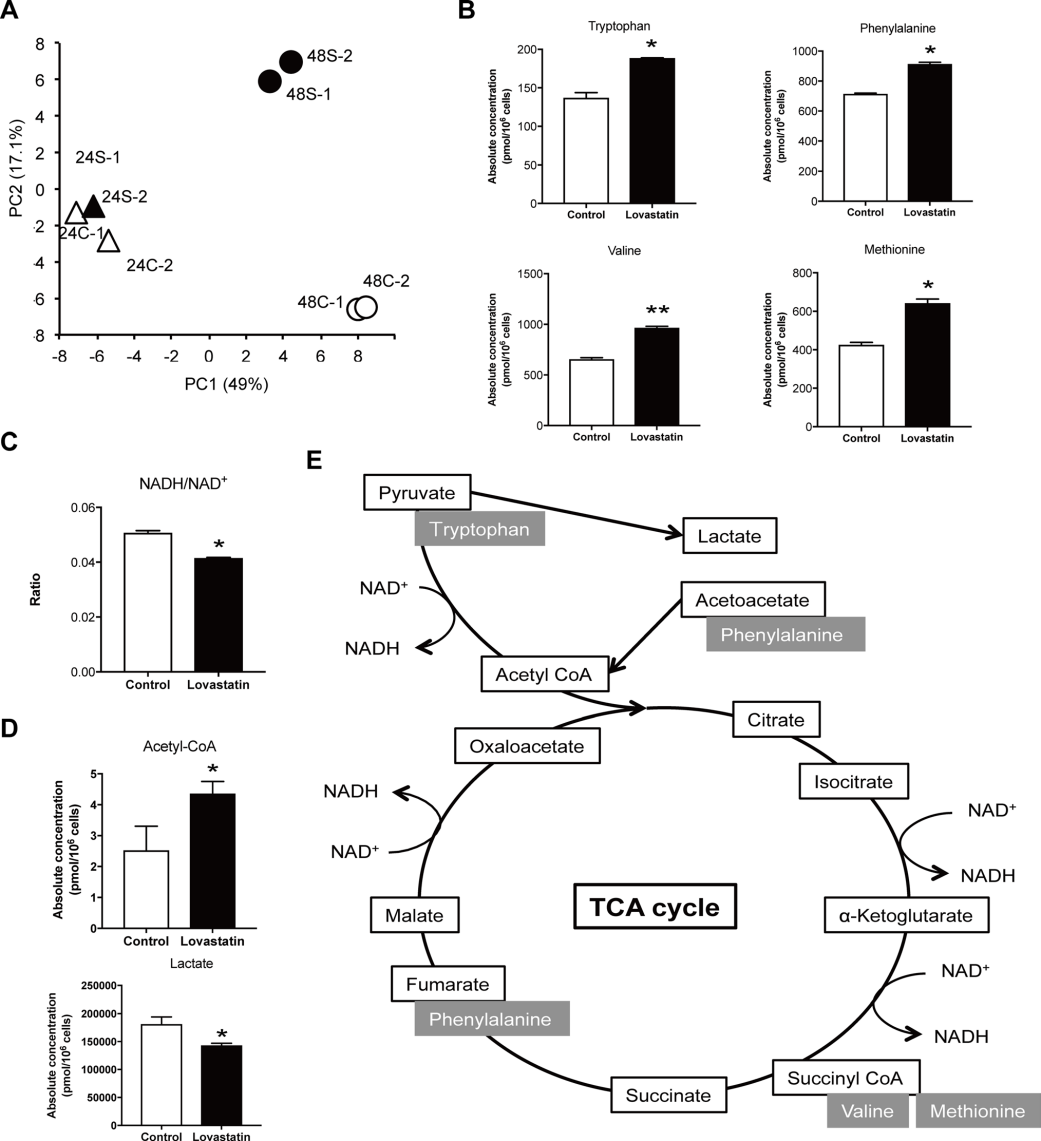
Lovastatin administration increased TCA cycle related metabolites and interfered glycolysis (**A**) Principal component (PC) analysis was conducted in order to compare the overall metabolomic profiles in harvested cells. 24C; control at 24 hours, 24S; lovastatin at 24 hours, 48C; control at 48 hours, 48S; lovastatin at 48 hours. (**B**) Increased metabolites, Tryptophan, Phenylalanine, Valine and Methionine, in lovastatin treated cells (*p < 0.05, **p < 0.01). (**C**) The ratio of NADH/NAD+ was calculated in lovastatin treated cells compared to control cells. It must be decreased when TCA cycle is activated (*p < 0.05). (**D**) The level of acetyl-CoA and lactate in lovastatin treated cells compared to control cells (*p < 0.05). (**E**) Graphs of increased metabolites by lovastatin treatment in TCA cycle.

The genetic basis of the antitumor effect of inhibition of the mevalonate pathway was already examined using microarray analysis. The results suggested that cell cycle control and chromosomal replication are involved in the mechanism [11]. Thus, we next focused on the aspects of metabolism of the antitumor effect. Metabolomics analysis was performed between control and statin-treated SKOV3 cells using capillary electrophoresis mass spectrometry. In principal component analysis, all 24-hour samples were grouped together, whereas 48-hour samples were separated into distinct groups of increased and decreased metabolites (Figure 4A). Metabolites with increases after statin treatment included those related to the tricarboxylic acid (TCA) cycle (Figure 4B). Nicotinamide adenine dinucleotide (NAD) is a coenzyme that is important in the electron transport system and is found in oxidized and reduced forms referred to as NAD^+^ and NADH, respectively. Upon activation of the TCA cycle, NAD^+^ is reduced to NADH, and then changed again to NAD^+^ to produce ATP; thus, totally the ratio of NADH/NAD^+^ is decreased when the TCA cycle is activated. Statin treatment also significantly reduced this ratio, which suggests that the statin activated the TCA cycle (Figure 4C). Furthermore, acetyl-CoA was significantly increased and lactate was significantly decreased in statin-treated cells (Figure 4D). In tumor cells, energy is basically generated by aerobic glycolysis instead of oxidative phosphorylation in mitochondria, giving the Warburg effect. Our findings suggest that statins may interfere with the Warburg effect and shift the cells to activation of the TCA cycle (Figure 4E).

### Inhibition of the mevalonate pathway reduces glutathione synthesis and changes sensitivity to anticancer agents

**Figure 5:**
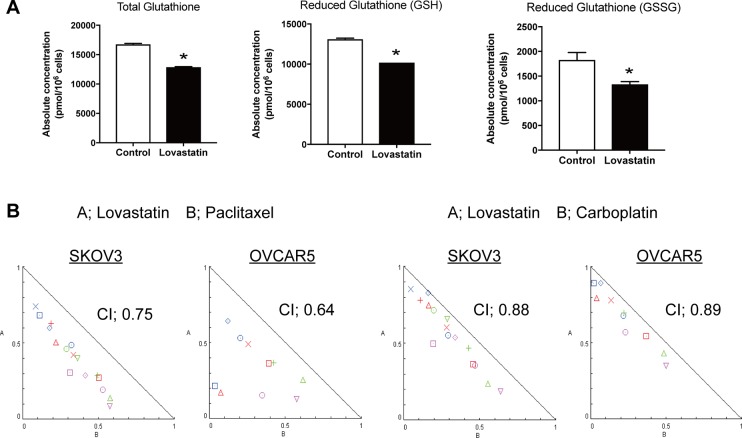
Lovastatin decrease Glutathione, and resulted to work with chemotherapeutic agents synergistically (**A**) The serum value of total, reduced and oxidized glutathione in lovastatin mediated cells (*p < 0.05). (**B**) Combination index was calculated in lovastatin treatment with paclitaxel or carboplatin.

Metabolomics analysis showed interesting findings for glutathione. Statin treatment significantly decreased total, reduced and oxidized glutathione (Figure 5A). Glutathione is an antioxidant that prevents damage to cellular components by reactive oxygen species (ROS) such as free radicals and peroxides. Glutathione levels are elevated in several human cancer tissues, including bone marrow, breast, colon, larynx and lung [17], whereas a glutathione level may induce autophagy and apoptosis, and increase the efficacy of chemotherapy [18–20]. We have previously shown that a statin induces apoptosis and autophagy, and we found that several other inhibitors of the mevalonate pathway induced autophagy in this study. To determine whether a statin changes sensitivity to chemotherapeutic agents, we checked the combination index of the statin with paclitaxel or carboplatin in SKOV3 and OVACAR5 cells. Both indexes were < 1.0, which suggests that the statin works synergistically with chemotherapeutic agents (Figure 5B).

## DISCUSSION

There are few studies of cancer from a perspective of metabolism. However, abnormalities in tumor suppressor genes are known to alter metabolism and are involved in malignant progression in the tumor microenvironment [21]. Metabolic changes are also implicated in tumor growth via effects on the cellular environment and epigenetic repression; therefore, abnormal metabolic changes are candidates as therapeutic targets [22, 23]. Analyses of metabolic changes in cancer using metabolomics have identified several previously unknown metabolic effects, and studies of the relationship between cancer and metabolism are becoming more common [24–26].

The mevalonate pathway results in biosynthesis of isopentenyl diphosphate and dimethylallyl diphosphate, which are starting materials for terpenoid and steroid synthesis, from acetyl-CoA, and isoprenoid intermediate metabolites including farnesyl pyrophosphate and geranylgeranyl pyrophosphate, which are required for prenylation of proteins. Ras and Rho family proteins play significant roles in cancer progression and are activated by prenylation; therefore, inhibition of the effects of these enzymes may account for the antitumor effect of inhibition of the mevalonate pathway [27, 28]. We have previously shown that statin inhibition of HMG-CoA reductase in this pathway has an antitumor effect on ovarian cancer *in vivo* [11], and the results of this study showed that all of the inhibitors of this pathway (6-fluoromevalonate inhibiting mevalonic kinase, bisphosphonate inhibiting farnesyl pyrophosphate, YM-5360 inhibiting squalene, Lonafarnib inhibiting farnesyltransferase related to farnesylation from farnesyl pyrophosphate, and GGTI-298 inhibiting geranylgeranyltransferase related to geranylgeranylation from geranylgeranyl pyrophosphate) induced autophagy and inhibited cell growth *in vitro*. Previous studies have shown reduced cell growth by inhibiting the mevalonate pathway, with induction of apoptosis and autophagy, but the mechanism of induction is unclear. This pathway is regulated by the Rb tumor suppressor gene, similarly to several other metabolic pathways [29], and inhibition of the mevalonate pathway may influence Rb function. Rb inhibits cell cycle progression and controls apoptosis and autophagy via a nontranscriptional mechanism [30], and effects on this mechanism may be involved in inhibition of cell growth.

Bisphosphonates incorporated in osteoclasts inhibit farnesyl pyrophosphate synthase, which results in reduced prenylation of proteins. Thus, these drugs inhibit posttranslational modification of proteins related to cell signaling, such as GTPase, and induce apoptosis of osteoclasts and inhibit bone turnover. In this study, alendronate, a nitrogen-containing second-generation bisphosphonate, had an inhibitory effect on ovarian cancer cell growth *in vitro* and an antitumor effect *in vivo*. Mogp-TAg transgenic mice formed tumors from around 6 wks, thus alendronate was administered from 3wks. Interestingly, alendronate suppressed the initiation of tumor compared to control, this suggest alendronate may work as chemopreventive drugs. In this study, alendronate was administered to mice daily at a dose of 15 mg/kg, and this dose was greatly exceeds commonly used doses in clinical setting. Although it is necessary to start from the consideration of the optimal dose in clinical trials, bisphosphonates may be useful as chemopreventive drugs for ovarian cancer through drug repositioning as well as statins.

Metabolomic analysis suggested a shift from the Warburg effect to activation of the TCA cycle in ovarian cancer cells. Cells generally efficiently produce energy (ATP) from glucose with oxidative phosphorylation in the TCA cycle and the electron transport chain in mitochondria by oxygen respiration. Under anoxic conditions, ATP is produced by anaerobic glycolysis (metabolic pathway to degrade glucose anaerobically and produce lactate) in the cytoplasm. In the Warburg effect in cancer cells, energy production by oxidative phosphorylation in mitochondria is decreased and energy production by anaerobic glycolysis in the cytoplasm is increased [31]. There are several possible reasons for conversion from oxidative phosphorylation, an efficient pathway of ATP production, to inefficient glycolysis in cancer cells: cancer cells can grow under hypoxic conditions due to a poor blood vessel supply, cell death (apoptosis) is unlikely to occur due to inhibition of oxidative phosphorylation in mitochondria, lactate inhibits the actions of immune cells, and acidification around cancer tissues facilitates invasion of cancer cells [32]. A recent hypothesis proposes that the Warburg effect results in enhanced production of NAD without the TCA cycle in order to increase biomass (lipids, nucleic acids, proteins) required for self-replication of cancer cells and minimize production of ROS, which are potent inhibitors of cell growth, because the pentose phosphate cycle branched from glycolysis is facilitated and nucleic acid, protein and fatty acid syntheses are increased under hyperglycemic conditions [33]. In cancer cells, the large change in metabolic conditions compared to normal cells is referred to as metabolic reprogramming [34]. In this study, statin inhibition of the mevalonate pathway seemed to activate the TCA cycle and return cells to metabolic conditions closer to those in normal cells, thus reducing the Warburg effect. Conventional anticancer agents target DNA replication and molecular targeted drugs block signals for cell growth. The results of this study suggest a new strategy for cancer treatment via metabolic regulation targeting cancer-specific metabolic abnormalities.

The potential antitumor effects of mevalonate pathway inhibitors, including statins and bisphosphonates, are supported by epidemiological and basic studies, including this study. Clinical trials will be performed soon to confirm these effects. However, there are several studies that do not support a causal relationship of statins with cancer incidence and mortality. These studies suggest that an antitumor effect of mevalonate pathway inhibitors is possible, but is unlikely to occur for all cancers. Given the pharmacological mechanism of statins and bisphosphonates, an antitumor effect of these drugs is likely in cancers with an activated mevalonate pathway. Therefore, inclusion criteria for subjects in whom an effect is expected should be established based on genetic tests prior to clinical trials. Molecular targeted drugs have also been developed for various cancers including ovarian cancer and its effects are highly expected, but there is concern that the national health expenditures will rise due to the cost from drug development. The findings in this study that existing drugs such as statin and bisphosphonate may act on chemopreventive for ovarian cancer may contribute to solve such social problems.

## MATERIALS AND METHODS

### Cell culture and cell proliferation assay

Two ovarian cancer cell lines, SKOV3 and OVCAR5, were purchased from American Type Culture Collection (ATCC). Both cell lines were cultured at 37°C under 5% CO_2_ in RPMI-1640 supplemented with 10% heat-inactivated fetal calf serum, penicillin (100 U/mL), and streptomycin (100 U/mL). Cell proliferation was evaluated using a tetrazolium salt (MTT) assay. Concentrations for 50% inhibition of growth (IC_50_) were calculated using Prism 7 (GraphPad Software, La Jolla, CA).

### Western blot analysis

Tumor tissues or cells were homogenized in lysis buffer (50 mmol/L Tris-HCL, pH 7.5, 150 mmol/L NaCl, 1% NP40) with Halt Protease Inhibitor Cocktail (1861278, Thermo Fisher Scientific). Protein concentrations in tissues or cell lysates were determined with a protein assay kit (Bio-Rad), using bovine serum albumin as a standard. Aliquots of protein lysate (30 mg) were separated by SDS-PAGE, and Western blot analyses were performed using standard procedures. Blots were developed using an Amersham ECL Western Blotting Detection Reagents kit (GE Healthcare UK), using primary antibodies LC3A (#4599), LC3B (#3868), and GAPDH (#5174; all from Cell Signaling Technology).

### Animal studies

Generation of the mogp-TAg transgenic mouse has been described elsewhere [14, 15]. Mice were housed and handled according to a protocol approved by the Johns Hopkins University Animal Care and Use Committee. The mogp-TAg genotype was confirmed by tail DNA extraction and polymerase chain reaction (PCR). PCR was performed using denaturation at 94°C for 30 s, followed by 30 cycles at 94°C for 15 s, 55°C for 30 s, 68°C for 45 s, and a final extension at 68°C for 5 min. The primer sequences were forward: GAA AAT GGA AGA TGG AGT AAA, and reverse: AAT AGC AAA GCA AGC AAG AGT. mogp-TAg mice were treated daily with 15 mg/kg alendronate sodium trihydrate (A4978, Sigma-Aldrich, St. Louis, MO), a bisphosphonate, in 0.5% methylcellulose by gastric intubation using disposable feeding tubes from 3 weeks of age until euthanasia at 8 weeks. Reproductive tracts were removed, weighed, formalin-fixed, and embedded in paraffin. Because tumor cells account for about 75% of the total mass of the female genital tract in untreated mice, tissue weight was used as an indicator of tumor burden.

### Metabolomics analysis

Analysis of intracellular metabolites was performed by the Carcinoscope analysis service (Human Metabolome Technologies (HMT) America, Boston, MA). Targeted quantitative analysis was performed on 13 samples of harvested cells using capillary electrophoresis mass spectrometry (CE-MS) in cation and anion analysis modes. A total of 116 metabolites (54 cations and 62 anions) involved in glycolysis, pentose phosphate pathway, tricarboxylic acid (TCA) cycle, urea cycle, and polyamine, creatine, purine, glutathione, nicotinamide, choline, and amino acid metabolisms were annotated based on the HMT metabolite database.

### Combination index analysis

The combination index (CI) of lovastatin with paclitaxel or carboplatin was calculated using Chou's combination index (CI) model in the CalcuSyn program [35]. CI is a quantitative measure of the degree of interaction between drugs: CI < 1 indicates synergism; CI = 1 denotes additive effects; and CI > 1 denotes antagonism.

### Analysis of plasma cholesterol and triglycerides

Mice were euthanized and blood was collected by intracardiac aspiration using a 1-mL syringe with a 25-gauge needle and placed in a microcentrifuge tube containing EDTA. Blood was centrifuged for isolation of plasma, and cholesterol and triglycerides were measured using standard clinical laboratory assays on a Roche Hitachi Cobas c701 analyzer (Roche Diagnostics).

### Statistical analysis

Statistical analyses were performed with Prism 7.0 GraphPad software. The Mann–Whitney *U* test was performed to assess tumor volume for vehicle- and lovastatin-treated groups. Specific analyses performed for each assessment are described in the results and figure legends. In all analyses, data were evaluated using a two-tailed test; *P* < 0.05 was considered statistically significant.
